# The role of complement components C1q, MBL and C1 inhibitor in pathogenesis of endometriosis

**DOI:** 10.1007/s00404-018-4754-0

**Published:** 2018-03-23

**Authors:** Justyna Sikora, Agnieszka Wróblewska-Czech, Marta Smycz-Kubańska, Aleksandra Mielczarek-Palacz, Anna Cygal, Andrzej Witek, Zdzisława Kondera-Anasz

**Affiliations:** 1 Department of Immunology and Serology, School of Pharmacy with the Division of Medical Analytics in Sosnowiec, Medical University of Silesia in Katowice, Jedności 8, 41-200 Sosnowiec, Poland; 2 Department of Gynecology and Obstetrics, School of Medicine in Katowice, Medical University of Silesia in Katowice, Medyków 14, 40-752 Katowice, Poland

**Keywords:** Complement components, Endometriosis, Peritoneal fluid

## Abstract

**Purpose:**

The purpose of the work was to evaluate possible associations between the complement components C1q, mannose-binding lectin (MBL) and C1 inhibitor (C1INH) with pathogenesis of endometriosis.

**Methods:**

Concentrations of C1q, MBL and C1INH were measured by ELISA in peritoneal fluid (PF) in 80 women with or without endometriosis.

**Results:**

Significantly higher PF levels of C1q, MBL and C1INH in women with endometriosis compared to control group were observed (*p* < 0.0001). A higher concentration of the studied parameter was found in PF of women at the early stage of the disease, as compared to women with advanced endometriosis (*p* < 0.0001).

**Conclusions:**

Our research suggests that in the peritoneal cavity in women with endometriosis there are abnormal regulations of both the classical and lectin pathways of the complement system. This can suggest impairments in purification of peritoneal cavity from ectopic endometrial cells and augmented local inflammation in endometriosis patients.

## Introduction

Endometriosis is a common gynecological condition that affects approximately 10–12% of female population in reproductive age and up to 60% of infertile women [1]. It is defined as the presence and growth of endometrial-like tissues ectopically, most commonly on the pelvic peritoneum and ovaries [2]. Although endometriosis was first described more than 150 years ago, the pathogenesis of the disease is still poorly understood. The retrograde menstruation, a widely accepted theory, explained the possibility of endometrial cells appearing in the peritoneal cavity, but did not explain why cells were living there [3]. Recently, studies have strongly suggested that local and systemic dysfunction of the immune system and inflammation must be involved in the survival of endometrial implants in ectopic localization [4]. In a physiological condition, well-functioning immune system mechanisms, eliminate endometrial cells from the peritoneal cavity. However, in women with endometriosis, it seems that the immune dysfunction and an altered peritoneal environment are associated with the reduced clearance of retrogradely transported endometrial fragments [5]. In the peritoneal fluid (PF) a high accumulation of peritoneal macrophages, regulatory T suppressor cells, NK cells and cytotoxic T lymphocyte with reduced killing capacity, cytokines, immunoglobulins (Ig) and inflammatory mediators can be found [6, 7].

One of the most important immune mechanisms taking part in the peritoneal clearance and inflammation is the complement system. The complement, a crucial component of the innate immune system, consists of over 30 small proteins which act as a cascade of proteases that activate each other in an enzymatic fashion, with effector mechanisms mediated by several specific cell receptors [8, 9]. The general function of the complement is to recognize microbial pathogens and other target cells and let them lyse. The complement cascade can be activated via one of three pathways: the classical pathway, the lectin pathway and the alternative pathway. These pathways are activated via different recognition molecules. The alternative complement pathway is initiated directly by the pathological organism. The classical pathway is started by the activation of C1q, by binding to ligands such as surface-bound IgM or IgG [10]. C1q is a plasma glycoprotein produced mainly by a wide range of immune cells including monocytes and macrophages, dendritic cells, and by epithelial and endothelial cells, as well as fibroblasts and trophoblasts [11]. The connection to the globular head domain of C1q with IgG or IgM containing immune complexes, causes an activation of C1r, which in turn activates C1s [8]. The lectin pathway is like the classical pathway. The cascade is initiated by binding of mannose-binding lectin (MBL) to mannose contained on the surface of the target cells. It is associated with various MBL-associated serine proteases (MASPs), which upon activation lead to the cleavage of C2 and C4 [12].

**Fig. 1:**
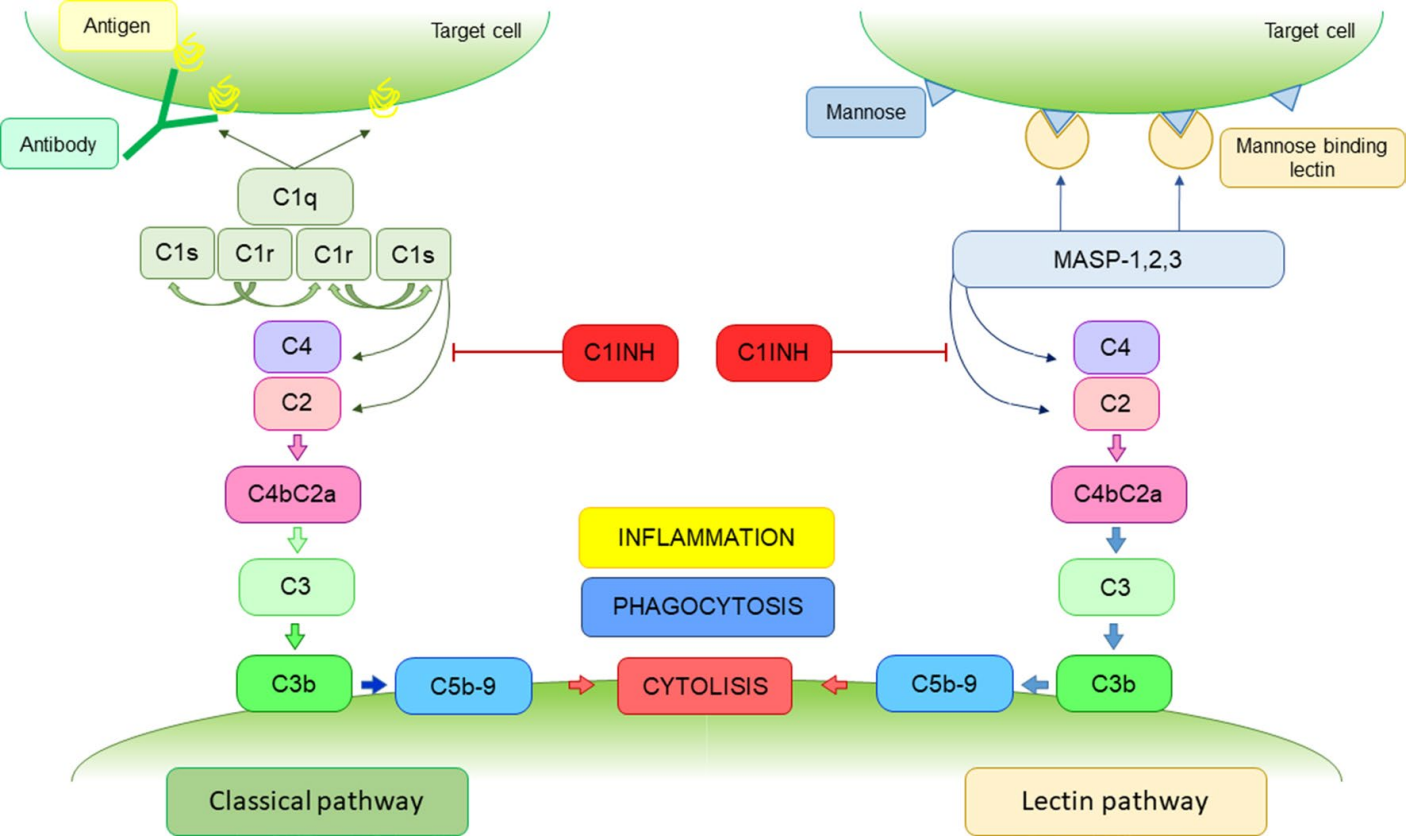
The complement system activation via classical and lectin pathways

The activated C1 complex and MBL cleave the C4 and C2 components in the classical and lectin complement cascade, resulting in the assembly of the C3 convertase (C4b2a) [13]. In all three pathways the sequence of reactions leads to the generation of protease—C3 convertase and finally a membrane attack complex. This cascade of reactions leads to the creation of a pore in the cell membrane of target cell which destroys its integrity and results in lysis of the cell [13]. The activation of the complement system is closely monitored at each stage by both activators and inhibitors. One such control agent is C1 inhibitor (C1INH). It is a circulating complement regulatory protein which can inactivate C1r, C1s, MASP-1 and MASP-2, thereby preventing/limiting complement activation via both the classical and the leptin pathway 9. The role of C1INH was shown in Fig. 1.

Recent data have linked disorders of the complement system activity and pathogenesis of endometriosis [14]. For this purpose, we decided to investigate the levels of C1q, MBL and C1 inhibitor in the peritoneal fluid of patients with endometriosis in relation to the stage of the disease.

## Materials and methods

### Study population

Eighty women aged between 21 and 49 (mean age 28.4 ± 4.3 years) undergoing laparoscopy for infertility were included in this study. All patients were admitted to the Clinic of Gynecology and Obstetrics of the Medical University of Silesia in Katowice, for diagnostic or therapeutic laparoscopy for infertility. Clinical data regarding the phase of menstrual cycle, obstetrical history, previous gynecological surgical procedures and the history of hormone use were also obtained by means of an appropriate questionnaire. All diagnostic and laparoscopic procedures were performed during the proliferative phase of the menstrual cycle. The women included in the study had a normal menstrual cycle and no other pelvic disorders, chronic circulatory, autoimmune or neoplastic disease and had not been taking any anti-inflammatory or immunomodulatory medications in the preceding 2 months.

Endometriosis was confirmed histologically in 60 women, aged 21–49 years (mean age ± SD 31.9 ± 7.0 years). The studied group, including early (stage I and II, *n* = 35) and advanced (stage III and IV, *n* = 25) endometriosis, were scored by the revised American Fertility Society (rAFS) classification [15]. The control group included 20 women, aged 23–46 years (mean age 30.8 ± 6.4 years) who had unexplained infertility but no evidence of endometriosis or inflammation in the peritoneal cavity. The study protocol was approved by the Ethics Committee of the Medical University of Silesia (KNW/0022/KB/123/14). All patients and control subjects who participated in this study signed an informed consent form.

### Collection and processing of peritoneal fluids samples

Peritoneal fluid (PF) samples were collected by aspiration after insertion of the laparoscope with a laparoscopic cannula directly from the Douglas’ cul-de-sac before any surgical intervention to avoid contamination of the fluid with blood. After aspiration, the samples were processed by centrifugation at 400×*g* for 10 min and stored at − 85 °C until the analysis.

### Complement components assay

PF levels of the studied parameters were measured by a standard cytokine-specific enzyme-linked immunosorbent assay (ELISA) using commercial kits: Enzyme-linked Immunosorbent Assay Kit for complement 1q (Cloud Clone Corp., Houston, USA) and RayBio^®^Human MBL ELISA Kit (RayBiotech, Norcross, USA). The sensitivity of the kits was approximately 32 pg/ml for C1q and 0.03 ng/ml for MBL. The level of C1 inhibitor was measured with MicroVue C1-Inhibitor Plus EIA test (Quidel Corporation, Hannover, Germany). The concentration of functional C1-INH in a given sample is reported as the percentage of the mean level in normal specimens. Concentrations greater than or equal to 68% of mean normal are considered normal.

### Statistical analysis

All results were presented as mean ± SD and were tested for normality of distribution by the Shapiro–Wilk test. Parametric data were analyzed using Student’s *t* test, whereas nonparametric data were analyzed according to Fisher’s exact test (ANOVA). Spearman’s rank correlation test was used to determine correlations, which were presented as a correlation coefficient (r). A *P* value ≤ 0.05 was considered as statistically significant. Analyses were performed with Statistica 12.0.

## Results

**Fig. 2:**
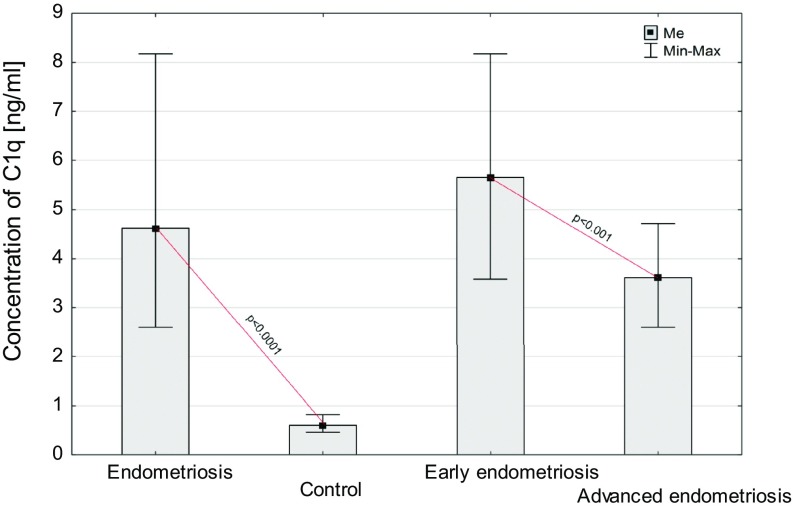
Concentration of C1q in peritoneal fluid of women with endometriosis and women from control group and women with early and advanced endometriosis

**Fig. 3:**
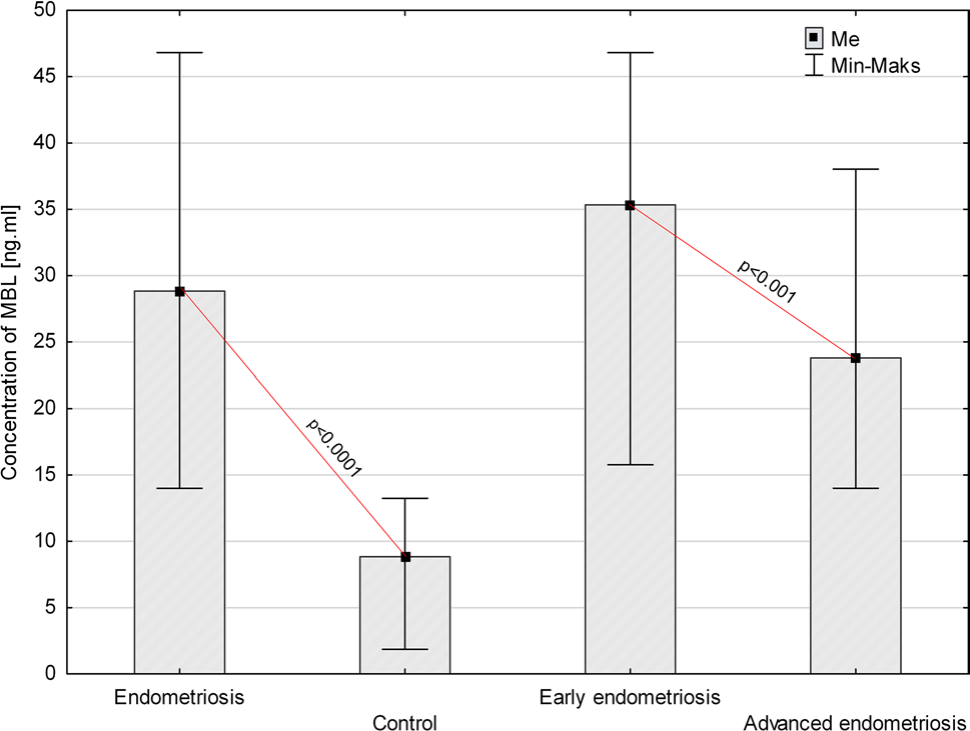
Concentration of MBL in peritoneal fluid of women with endometriosis and women from control group and women with early and advanced endometriosis

**Fig. 4:**
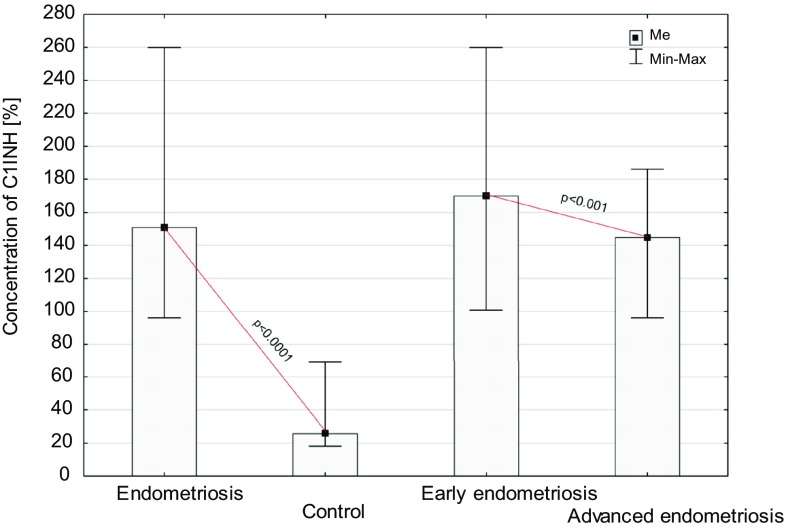
Concentration of C1INH in peritoneal fluid of women with endometriosis and women from control group and women with early and advanced endometriosis

The concentrations of the complement components: C1q, MBL and C1 inhibitor in PF of women with endometriosis and the control group are shown in Figs. 2, 3 and 4.

The PF C1q level was significantly higher in women with endometriosis than in the controls (*p* < 0.0001). Moreover, concentrations of the studied parameter in PF of women with early and advanced disease increased compared to the control group (*p* < 0.0001 for early and *p* < 0.001 for advanced stage). A higher concentration of the studied parameter was found in PF of women at the early stage of the disease, as compared to women with advanced endometriosis (*p* < 0.001).

The concentration of MBL in PF was significantly higher in women with endometriosis than in the control group (*p* < 0.0001). Similarly, the level of the factor in the PF of women with early and advanced disease was higher than in the PF of control group (*p* < 0.0001). A higher concentration of MBL was found in the PF of women at the early stage of the disease compared to women with advanced endometriosis (*p* < 0.001).

The concentration of C1INH in the PF was significantly higher in women with endometriosis than in the control group (*p* < 0.0001). Similarly, the level of inhibitor in the PF of women with early and advanced disease was higher than in the PF of the control group (*p* < 0.0001). A higher concentration of the studied parameter was found in the PF of women at the early stage of the disease compared to women with advanced endometriosis (*p* < 0.001).

**Fig. 5:**
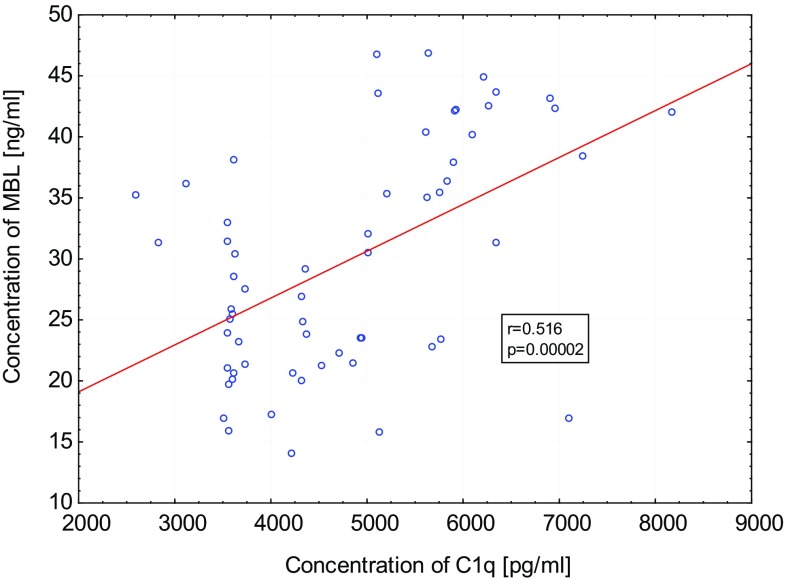
Correlation between concentrations of C1q and MBL in peritoneal fluid of women with endometriosis

Because all studied parameters are involving each other, we evaluated the connection between the concentrations of those parameters. In the PF of women with endometriosis, we observed statistically significant positive correlation between C1q and MBL levels (*p* < 0.0001, *r* = 0.516) (Fig. 5). There were not any significant correlations between C1q and C1INH and MBL and C1INH in the PF of affected women.

We then calculated the ratio of the concentrations of C1q and C1INH and MBL and C1INH, taking into consideration that C1INH is required for inhibition of the classical and lectin starting way of complement activation. The PF C1q/C1INH ratio in endometriosis was significantly higher compared to the control (*p* < 0.0001). Likewise, the differences between the values of ratios in early and advanced disease (*p* < 0.0001) were observed. However, MBL/C1INH ratio in the endometriosis group was significantly lower than in the control (*p* < 0.0001). At an early stage of the disease the value of ratio was significantly higher than in advanced endometriosis (*p* < 0.01). All these results are shown in Table 1.

**Table 1:** Peritoneal C1q/C1INH and MBL/C1INH ratios in women with endometriosis and control group

Ratio	Endometriosis			Control group (*n* = 20)
	All (*n* = 60)	Early (*n* = 35)	Advanced (*n* = 25)	
C1q/C1INH	0.03 ± 0.01*	0.04 ± 0.01*,****	0.02 ± 0.006**	0.02 ± 0.007
MBL/C1INH	0.20 ± 0.06*	0.22 ± 0.08***,*****	0.18 ± 0.05*	0.28 ± 0.09

Results are expressed as mean ± SD

*p < 0.0001 compared to control group

**NS compared to control group

***p < 0.001 compared to control group

****p < 0.0001 compared to advanced endometriosis

*****p < 0.01 compared to advanced endometriosis

## Discussion

Dysregulation of the immune system in endometriotic milieus has been considered to play a pivotal role in the pathogenesis of endometriosis. However, the role of the immune system in the pathogenesis of the disease is not completely understood. Physiologically, ectopic endometrial tissue is eliminated from the peritoneal cavity by the immune system and dysregulation of clearance mechanisms can predispose to implantation of ectopic cells and progression of endometriosis [16]. The complement system, the part of the innate immune system, is involved in a variety of inflammatory reactions and supplements the humoral immunity [17].

In our study, we analyzed the concentration of C1q, MBL and C1 inhibitor in PF of women with endometriosis and control group. The analysis of results showed a higher concentration of all studied parameters in PF of affected women in comparison to control. Moreover, their levels were increased in women with an early stage of disease compared to advanced endometriosis. C1q and MBL are first factors which started the classical and lectin activation of the complement system. Their concentration and activity are important to correct the activation of both routes and then lysis of target cells. Our results showed higher levels of both complement components, so it may suggest that the activation of complement in women with endometriosis is correct. Under normal circumstances, the complement activation is tightly controlled by several regulatory proteins to minimize host tissue damage. However, one step later, in complement cascade, there is C1INH, and a higher concentration of it was found. This, in turn, may indicate the existence of additional factors that may affect the inhibition of complement activation and influence C1INH secretion.

In the available literature, there is no information about the role of C1q and C1INH in the formation and development of endometriosis. However, our results suggest that both factors may play an important role in the pathogenesis of the disease. It may be related with their properties, because locally synthesized C1q has been shown to induce a plethora of biological functions through the induction of immunomodulatory molecules by an autocrine- or paracrine- mediated signaling in a manner that mimics those of the tumor necrosis factor (TNF)-α. These include the recognition of pathogen- and danger-associated molecular patterns, phagocytosis, angiogenesis, apoptosis and induction of cytokines or chemokines that are important in modulating the inflammatory response. The functional convergence between C1q and TNF-α in turn is attributed to their shared genetic ancestry [18]. Moreover, studies have shown that C1q can play an important role in other diseases, including autoimmunological diseases [19]. Immunological complexes of IgM, IgG are needed to start the activation of C1 complex [8]. Recently, studies have reported that women with endometriosis demonstrate an increase in B-cell activation and systemic antibody production [20]. It seems probable that the complex of immunoglobulins and ectopic endometrial cells may initiate a classical pathway in endometriosis, especially in early stages of disease. Our results have also shown a higher level of MBL, which is the first component of lectin way. Our MBL results disagree with previously published studies, in which the concentration and total amount of peritoneal MBL does not differ in patients with and without endometriosis [21]. Moreover, there were no differences in the serum MBL levels in patients with or without endometriosis, which suggested that lectin could be involved in the modulation of inflammatory responses, but it did not seem to take part in endometriosis pathogenesis [22, 23]. Unfortunately, we do not have an explanation why there is such a difference in the MBL concentration in our results and the results of Kruse et al.’s [21]. This may be related to the test used to measure the concentration or even the admixture of blood in the test sample. Additionally, the greatest changes in the immune system are localized within the peritoneal cavity, and changes in peripheral blood may therefore be non-detectable.

More so the research of other authors also indicates the activation of the complement system. Despite such an important role of the complement system in the pathogenesis of the disease, there is not much research on this subject. Most studies analyzed components C3 and C4, which are common for all three pathways [24, 25]. These were one of the first parameters evaluating the immune system, which were studied in women with endometriosis. They showed that the complement components C3c and C4 were significantly increased in PF of patients with endometriosis. Moreover, both C3 and C4 components revealed a higher activity in women with endometriosis as compared to those without this disease [26]. Current research also confirms the role of C3 in pathogenesis of the disease. However, the complement C3 had a higher PF levels in the luteal than in the follicular phase of the menstrual cycle in both infertile and fertile patients with endometriosis [27]. It may be related to the fact that the complement was equally likely to be found in proliferative, secretory, menstrual, or inflammatory endometrium. The endometrial complement was found less frequently in patients with severe endometriosis as compared with those with the mild form of it [28]. However, it should be remembered that these complement components belong to acute phase proteins and their elevated levels may not only correlate with impairments associated with endometriosis, especially when the test is performed in peripheral blood.

So, why does the complementary system not lyse the ectopic endometrial cells? The answer may be to evaluate the role of the complement activator regulators. One of the reasons of this situation can be an overactivation of complement regulators, including C1 inhibitor. This factor has been shown to inhibit the complement safely and is now being investigated in a variety of clinical conditions [18]. However, an impairment in the activity and concentration of C1INH can cause such diseases as angioedema, which is defined as a local, noninflammatory, self-limiting edema or the systemic lupus erythematosus [29, 30]. In the serum of 95% of patients with angioedema the level of C4 is low. These phenomena are due to the properties of the C1r subcomponent of C1, factor XII, and the bimolecular complex of prekallikrein with high-molecular-weight kininogen. Purified C1r auto-activates in physiologic buffers, activates C1s, which in turn depletes C4. This occurs when C1 inhibitor is deficient [31]. Perhaps, in the case of endometriosis, an over-production of C1INH may prevent classical and lectin activation of the complement, which may confirm the findings of other investigators demonstrating high C4 levels in the peritoneal dialysis fluid of women with endometriosis. However, confirmation of this hypothesis requires further research. The C1q/C1INH and MBL/C1INH ratios can contribute to better understanding of the relationships between the studied parameters. Differences in both ratios in women with and without endometriosis might suggest impairments in the activation and regulation of classical and lectin way of the complement system. However, the C1q/C1INH ratio was higher in endometriosis than in the control group. In turn, MBL/C1INH ratio was lower in endometriosis as compared to control. This might mean a more active classic path in these patients. Regardless of the way, aberrations of both ratios can result in the impairment of immune homeostasis in the peritoneal cavity in women with endometriosis.

In conclusion, our study reveals that there are changes in the regulation of the complement activation that leads to a chronic inflammation in endometriosis. Our research suggests that in the peritoneal cavity in women with endometriosis there are abnormal regulations of both the classical and lectin pathway of the complement system. However, further investigation is needed to completely explain the role of the complement system and the complement regulation mechanisms in pathogenesis of endometriosis.
